# How Psychological Capital and Academic Burnout Shape Short‐Video Addiction: A Latent Profile Analysis of Heterogeneity

**DOI:** 10.1002/brb3.71595

**Published:** 2026-07-14

**Authors:** Hongning Lin, Haiyu Xiong, Huaqiang Liu, Zhensong Lan, Qing Wang, Juan Cui, Siyun Liu, Yibing Guo

**Affiliations:** ^1^ School of Teacher Education Hechi University Yizhou China; ^2^ School of Humanities and Social Sciences Guangxi Medical University Nanning China; ^3^ School of Law and Public Administration Yibin University Yibin China; ^4^ School of Sociology and Humanities Jiangxi University of Finance and Economics Nanchang China; ^5^ School of Humanities Arts and Design Guangxi University of Science and Technology Liuzhou China

**Keywords:** academic burnout, adolescence, latent profile analysis, self‐control, short‐video addiction, psychological capital

## Abstract

**Background:**

Short‐video addiction among adolescents is a growing concern, yet existing research has largely adopted variable‐centered approaches that assume homogeneity.

**Aim:**

This study aimed to identify distinct stress–resource profiles based on psychological capital and academic burnout and to examine differences in self‐control and short‐video addiction across profiles, along with predictors of profile membership and the interactive effect of psychological capital and academic burnout on addiction.

**Methods:**

A cross‐sectional survey was conducted among 1719 Chinese middle school students (53.1% male). Latent profile analysis (LPA) was used to identify profiles. Differences in self‐control and addiction were tested with ANOVA, predictors with multinomial logistic regression, and the interaction with multiple linear regression.

**Results:**

Three profiles emerged: Adaptive (57.6%), Maladaptive (14.5%), and Advantaged (27.9%). The Advantaged profile showed the highest self‐control and the lowest addiction, whereas the Maladaptive profile showed the lowest self‐control. Post‐hoc tests revealed no significant difference in addiction between the Adaptive and Maladaptive profiles (p = 0.351), but both showed significantly higher addiction than the Advantaged profile. Female adolescents were less likely to belong to the Advantaged profile *(OR* = 0.75); a higher grade reduced odds of Maladaptive (OR = 0.86) and Advantaged (*OR* = 0.82) profiles; and left‐behind status increased odds of the Advantaged profile (OR = 1.90). Psychological capital moderated the burnout–addiction relationship (*B* = 0.035, *p* = 0.005); notably, the association strengthened as psychological capital increased, challenging the view of psychological capital as universally protective.

**Conclusion:**

Adolescents exhibit heterogeneous stress–resource profiles with distinct patterns of self‐control and addiction. Person‐centered approaches can inform tailored interventions, particularly by addressing both resource deficits and the complex role of psychological capital in coping processes.

## Graphical abstract

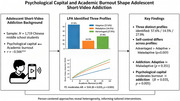

## 1 Introduction

Short‐video platforms have rapidly become a dominant form of entertainment and social interaction among adolescents, yet their immersive, algorithm‐driven design also poses significant risks of excessive use and addiction (Yang et al. 2023). The unique characteristics of short videos, such as brevity, high entertainment value, and personalized recommendations, may create a “filter bubble” that reinforces passive consumption and diminishes active self‐regulation (Wu et al. 2024; Huang et al. 2023). Consequently, understanding the antecedents of short‐video addiction is of critical importance.

Previous research has identified academic pressure, self‐control, and psychological capital as important correlates of adolescent internet‐related addictions (Zhang and Liu 2019; Song and Park 2022; Zewude et al., 2025). Academic burnout, defined as psychological and physical exhaustion resulting from prolonged academic stress (Sun et al. 2024), may drive adolescents to turn to short videos as a coping mechanism to escape from school‐related pressure (Guo and Chai 2024; Mu et al. 2022). Psychological capital, comprising self‐efficacy, resilience, hope, and optimism (Luthans et al. 2006), serves as a protective resource that enables individuals to adopt positive coping strategies and resist addictive behaviors (Wang and Qian 2025; Xiong et al. 2020). Moreover, recent studies have found that resilience and self‐control sequentially mediate the link between personality traits and short‐video addiction (Wu et al. 2024), suggesting a complex interplay between psychological resources and self‐regulation.

The Conservation of Resources (COR) theory (Hobfoll, 1989) provides a useful framework for understanding these dynamics. COR theory posits that individuals strive to protect and accumulate resources; when resources are threatened or depleted, stress ensues, and individuals may engage in maladaptive behaviors to cope. Importantly, COR theory also emphasizes resource gain spirals (where initial resources beget further resources) and resource loss spirals (where initial losses lead to further losses). In the academic context, adolescents with high psychological capital and low academic burnout may be situated in a resource gain spiral, whereas those with low psychological capital and high burnout may be trapped in a resource loss spiral. The combination of high academic burnout (a stressor) and low psychological capital (a resource deficit) may create a particularly vulnerable profile for short‐video addiction.

Self‐control, the ability to regulate impulses and resist temptations, has been consistently linked to behavioral addictions (Brevers and Turel 2019). Specifically, self‐control failure has been identified as a proximal predictor of problematic TikTok use (Miedzobrodzka et al. 2024). Individuals with high self‐control can better manage their short‐video use, whereas those with low self‐control are more susceptible to compulsive viewing (Zhan and Ren 2012). Recent research has confirmed that self‐control serves as a key protective factor against short‐video addiction, mediating the effects of various antecedents (He et al. 2024). However, it remains unclear whether self‐control operates differently across subgroups of adolescents who vary in their profiles of stress and resources.

While the independent effects of academic burnout, psychological capital, and self‐control on addiction are well documented, the field has largely relied on variable‐centered approaches that assume homogeneity across individuals. This assumption may obscure important heterogeneity in how adolescents combine stress and resource characteristics. Recently, person‐centered methods such as latent profile analysis (LPA) have been used to identify subgroups of individuals who share similar patterns across multiple indicators (Zhou et al. 2020; Zeng et al. 2021). For example, teacher studies have identified distinct profiles of work and family balance (Zeng et al. 2021). Yet, no study to date has applied LPA to examine the combined role of academic burnout and psychological capital in adolescent short‐video addiction, nor has it explored whether self‐control differs meaningfully across such profiles.

The present study aims to fill this gap by: (1) identifying distinct stress–resource profiles based on academic burnout and psychological capital among Chinese adolescents using LPA; (2) examining differences in self‐control and short‐video addiction across these profiles; (3) exploring demographic predictors of profile membership such as gender, grade, and left‐behind status; and (4) testing the interactive effect of psychological capital and academic burnout on short‐video addiction.

While psychological capital is generally considered a protective factor that mitigates addictive behaviors (Wang and Qian 2025; Xiong et al. 2020), some studies suggest that its role may be more complex. According to COR theory, individuals with abundant resources such as high psychological capital may be more motivated to actively invest those resources to cope with stressors, potentially leading to deliberate but excessive engagement with accessible digital tools like short videos (Luthans et al. 2007). Under conditions of high academic burnout, such individuals might intentionally use short videos as a form of ‘strategic escapism’ to restore positive affect or regain a sense of control (Hobfoll 1989). However, given the highly engaging and algorithm‐driven nature of short‐video platforms, this intentional use may escalate into habitual overuse, thereby strengthening the link between burnout and addiction. Conversely, individuals with low psychological capital may lack the cognitive and emotional resources to engage in such strategic coping, resulting in a weaker association between burnout and addiction. Thus, we hypothesize that psychological capital moderates the relationship between academic burnout and short‐video addiction.

Based on COR theory and prior empirical findings, we hypothesize that:

**H1**. *Adolescents can be classified into distinct stress–resource profiles based on their levels of academic burnout and psychological capital*.
**H2**. *The identified profiles will differ significantly in self‐control and short‐video addiction, with the maladaptive profile showing the lowest self‐control and highest addiction*.
**H3**. *Demographic factors such as gender, grade, and left‐behind status will predict profile membership*.
**H4**. *Psychological capital moderates the relationship between academic burnout and short‐video addiction*.

## 2 Methods

### 2.1 Participants

A cluster sampling method was used to recruit participants from October 2022 to February 2024. A total of 1765 middle school students from seven schools across five Chinese provinces (Henan, Shanxi, Guizhou, Shandong, and Jiangxi) were initially surveyed. Inclusion criteria were as follows (see Table 1): (1) age between 11 and 18 years; (2) voluntary participation; (3) ability to read and understand the questionnaire. After excluding invalid responses (e.g., patterned responses, logically inconsistent answers, or more than four consecutive missing items), 1719 valid questionnaires were retained (effective response rate = 97.4%). The final analytic sample consisted of 880 males (51.2%) and 833 females (48.8%). Grade distribution was as follows: seventh grade (*n* = 310, 18.0%), eighth grade (*n* = 275, 16.0%), ninth grade (*n* = 352, 20.5%), tenth grade (n = 284, 16.5%), eleventh grade (*n* = 261, 15.2%), and twelfth grade (*n* = 237, 13.8%). Regarding left‐behind status, 290 participants (16.9%) were left‐behind children (i.e., children who have been left in their hometowns by one or both parents migrating for work). The study was approved by the Ethics Committee of Yibin University (Approval No. 2023101001E) and adhered to the principles of the Declaration of Helsinki. Informed consent was obtained from all participants and their guardians.

**TABLE 1:** Demographic characteristics of the Final Analytic Sample (*N* = 1719).

Characteristic	*n*	%
Gender		
Male	880	51.2
Female	833	48.8
Grade		
Seventh	310	18.0
Eighth	275	16.0
Ninth	352	20.5
Tenth	284	16.5
Eleventh	261	15.2
Twelfth	237	13.8
Left‐behind status		
Yes	290	16.9
No	1420	83.1

*Note*: The age variable was not collected; all participants were aged 11–18 years per the inclusion criteria. Gender and left‐behind coding are based on the original questionnaire categories.

### 2.2 Measures

Demographic characteristics were collected using a self‐developed questionnaire, including gender (0 = male, 1 = female), grade (from seventh to twelfth), and left‐behind status (0 = no, 1 = yes).

Academic burnout was assessed using the Academic Burnout Self‐Assessment Scale developed by Dai (2015). The scale consists of 14 items across three dimensions: physical and mental exhaustion, academic detachment, and low sense of achievement. Items were rated on a 5‐point Likert scale (1 = strongly disagree, 5 = strongly agree), with higher scores indicating higher levels of academic burnout. Scores were calculated as the mean of all items (range: 1–5). In this study, the Cronbach's α coefficient for the scale was 0.88.

Psychological capital was measured using the Psychological Capital Scale developed by Ye and Fang (2015). The scale comprises 20 items covering four dimensions: self‐efficacy, resilience, hope, and optimism. Items were rated on a 6‐point Likert scale (1 = strongly disagree, 6 = strongly agree), with higher scores reflecting greater psychological capital. Scores were calculated as the mean of all items (range: 1–6). In this study, the Cronbach's α coefficient was 0.87.

Self‐control was assessed using the Self‐Control Scale developed by Morean et al. (2014) and adapted for Chinese samples by Tan and Guo (2008). The scale contains 13 items designed to evaluate individuals’ ability to regulate behaviors and emotions. Items were rated on a 5‐point Likert scale (1 = strongly disagree, 5 = strongly agree), with higher scores indicating stronger self‐control. Scores were calculated as the mean of all items (range: 1–5). In this study, the Cronbach's α coefficient was 0.83.

Short‐video addiction was measured using the Short Video Addiction Scale developed by You et al. (2022). The scale consists of 12 items assessing adolescents’ dependence on and addictive tendencies toward short‐video platforms. Items were rated on a 5‐point Likert scale (1 = strongly disagree, 5 = strongly agree), with higher scores indicating greater severity of addiction. Scores were calculated as the mean of all items (range: 1–5). In this study, the Cronbach's α coefficient was 0.81.

For all scales, reverse coding was performed for negatively worded items according to the original scale instructions. Detailed information about reverse‐coded items and scoring procedures can be found in the respective scale citations.

### 2.3 Data Analysis

Data analyses were conducted using SPSS 26.0, R 4.4.3 (with packages mclust, dplyr, tidyr, ggplot2, and nnet), and Mplus 8.3. Descriptive statistics and Pearson correlations were computed for all study variables. To test for common method bias, Harman's single‐factor test was conducted; the unrotated factor analysis revealed that the first factor accounted for 24.6% of the total variance, below the recommended threshold of 40% (Podsakoff et al. 2003), indicating that common method bias was not a serious concern.

Latent profile analysis (LPA) was performed using the mclust package in R to identify subgroups of adolescents based on standardized scores of psychological capital and academic burnout. LPA is a person‐centered method widely used to identify homogeneous subgroups within heterogeneous populations (Šakan et al., 2024). Models with 1 to 4 profiles were estimated using the VVI model (diagonal variance with varying volume and shape). The optimal number of profiles was determined by comparing the Bayesian Information Criterion (BIC), Integrated Completed Likelihood (ICL), the adjusted Lo‐Mendell‐Rubin (aLMR) test, and the Bootstrap Likelihood Ratio Test (BLRT), with a minimum of 5% of the sample in each profile. Lower BIC and ICL values, a non‐significant aLMR (p > 0.05) indicating no improvement beyond the current solution, and a significant BLRT (p < 0.05) were considered as criteria (Nylund et al. 2007).

After profile extraction, one‐way ANOVA with Bonferroni post‐hoc tests was used to examine differences in self‐control and short‐video addiction across profiles. Multinomial logistic regression was applied to investigate the effects of gender, grade, and left‐behind status on profile membership. Finally, multiple linear regression was conducted to test the interactive effect of psychological capital and academic burnout on short‐video addiction. Statistical significance was set at *p* < 0.05 (two‐tailed).

No a priori sample size calculation was performed. Instead, we followed established guidelines for latent profile analysis, which recommend a minimum sample size of 500 for stable profile extraction (Nylund et al. 2007). Our final sample of 1719 participants far exceeds this benchmark, providing adequate statistical power.

Classification quality was evaluated using Entropy and average posterior probabilities.

## 3 Results

### 3.1 Descriptive Statistics and Correlations

Means, standard deviations, and Pearson correlations for all study variables are presented in Table 2. Psychological capital was significantly negatively correlated with academic burnout (*r* = −0.555, *p* < 0.001) and short‐video addiction (*r* = −0.177, *p* < 0.001) and positively correlated with self‐control (*r* = 0.447, *p* < 0.001). Academic burnout was positively correlated with short‐video addiction (*r* = 0.301, *p* < 0.001) and negatively correlated with self‐control (*r* = −0.355, *p* < 0.001). Self‐control was negatively correlated with short‐video addiction (*r* = −0.190, *p* < 0.001).

**TABLE 2:** Means, standard deviations, and correlations among study variables.

Variable	*M*	*SD*	1	2	3	4
1. PC	3.793	0.779	—			
2. AB	2.723	0.636	−0.566***	—		
3. SC	2.948	0.564	0.445***	−0.432***	—	
4. SVA	2.617	0.713	−0.174***	0.303***	−0.234***	—

*Note*: N = 1719. All variables are item‐mean scores. Abbreviations: AB = Academic Burnout, PC = Psychological Capital, SC = Self‐Control, SVA = Short‐Video Addiction. ***p < 0.001.ss

### 3.2 Latent Profile Analysis: Identification of Stress–Resource Profiles

LPA was conducted using standardized scores of psychological capital and academic burnout as indicators. Models with 1 to 4 profiles were estimated. Fit indices are shown in Table 3. The 3‐profile solution was selected based on lower BIC and ICL values, a non‐significant aLMR test for the 4‐profile solution (*p* = 0.321), a significant BLRT for the 3‐profile solution (*p* < 0.001), interpretability, and a minimum class size > 5% (BIC = −9083.60, ICL = −9732.88). The three profiles comprised 990 (57.6%), 250 (14.5%), and 479 (27.9%) adolescents, respectively.

**TABLE 3:** Fit indices and classification quality for Latent Profile Models.

No. of profiles	BIC	ICL	aLMR (p)	BLRT (p)	Class sizes (%)	Entropy	Avg. Post. Prob.
1	−9696.45	−9696.45	—	—	1719 (100%)	—	—
2	−9402.31	−9720.67	< 0.001	< 0.001	1159 / 560	—	—
**3**	**−9083.60**	**−9732.88**	**0.068**	**< 0.001**	**990 (57.6%)/250 (14.5%)/479 (27.9%)**	**0.368**	**0.841**
4	−9110.54	−9850.24	0.321	<0.001	1017 / 177 / 413 / 112	—	—

Abbreviations: aLMR = adjusted Lo‐Mendell–Rubin test, Avg. Post. Prob. = average posterior probability, BIC = Bayesian Information Criterion; BLRT = Bootstrap Likelihood Ratio Test, ICL = Integrated Completed Likelihood. Bold indicates the selected model. Entropy values > 0.8 are generally considered good, though the present Entropy of 0.368 suggests some overlap between profiles—this is acknowledged as a limitation.

### 3.3 Characteristics and Naming of the Profiles

**FIGURE 1:**
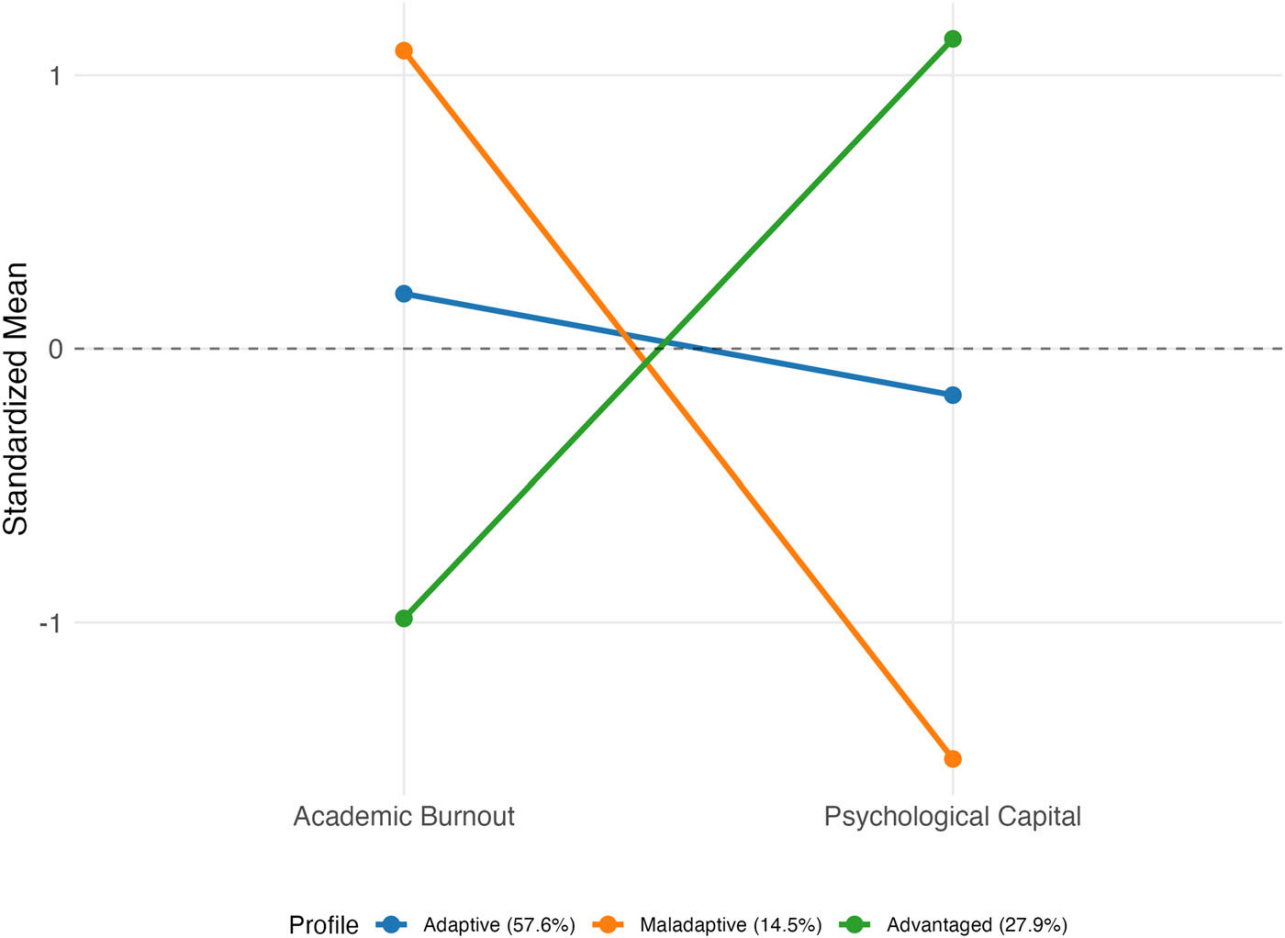
Standardized mean profiles of psychological capital and academic burnout across the three latent profiles.

The standardized means of psychological capital and academic burnout for each profile are displayed in Table 4 and visualized in Figure 1. Based on these patterns, the profiles were labeled as follows: Adaptive (Class 1, 57.6%): Slightly below‐average psychological capital (*z* = −0.17) and slightly above‐average academic burnout (*z* = 0.20), close to the overall mean. Maladaptive (Class 2, 14.5%): Very low psychological capital (*z* = −1.50) and very high academic burnout (*z* = 1.09). Advantaged (Class 3, 27.9%): Very high psychological capital (*z* = 1.13) and very low academic burnout (*z* = −0.99).

**TABLE 4:** Standardized means of psychological capital and academic burnout by profile.

Profile	*N*	%	Psychological capital (*z*)	Academic burnout (*z*)
Adaptive	990	57.6	−0.17	0.201
Maladaptive	250	14.5	−1.499	1.090
Advantaged	479	27.9	1.133	−0.985

### 3.4 Differences in Self‐Control and Short‐Video Addiction Across Profiles

One‐way ANOVA revealed significant differences among the three profiles in both self‐control (*F* (2, 1716) = 189.6, p < 0.001, η^2^ = 0.181) and short‐video addiction (*F* (2, 1716) = 39.9, *p* < 0.001, η^2^ = 0.044). Bonferroni post‐hoc tests indicated that for self‐control, the advantaged profile scored highest, followed by the adaptive profile, with the maladaptive profile scoring lowest (all *p* < 0.001). For short‐video addiction, the advantaged profile showed significantly lower addiction than both the adaptive and maladaptive profiles (*p* < 0.001), whereas the adaptive and maladaptive profiles did not differ significantly (*p* = 0.999). Descriptive statistics are presented in Table 5.

**TABLE 5:** Differences in self‐control and short‐video addiction across profiles.

Profile	Self‐control (M ± SD)	Short‐video addiction (M ± SD)
Adaptive (*n* = 990)	2.87 ± 0.45	2.70 ± 0.66
Maladaptive (*n* = 250)	2.59 ± 0.63	2.76 ± 0.85
Advantaged (*n* = 479)	3.30 ± 0.55	2.38 ± 0.69
*F*	189.6***	39.9***
η^2^	0.181	0.044
Post‐hoc (Bonferroni)	Advantaged > Adaptive > Maladaptive	Advantaged < Adaptive, Advantaged < Maladaptive (Adaptive = Maladaptive, p = 0.351)

*Note*: *** p < 0.001. Abbreviations: SC = Self‐Control, SVA = Short‐Video Addiction.

Figure 3 visualizes the differences in self‐control and short‐video addiction across the three profiles.

### 3.5 Predictors of Profile Membership: Multinomial Logistic Regression

Multinomial logistic regression was conducted to examine the effects of gender, grade, and left‐behind status on profile membership, with the adaptive profile as the reference group (see Table 6). The model showed good fit (residual deviance = 3103.78, AIC = 3119.78). Female adolescents were less likely than males to belong to the advantaged profile (OR = 0.75, *p* = 0.013), but gender did not significantly differentiate the maladaptive profile from the adaptive profile. Higher grade was associated with lower odds of being in both the maladaptive (OR = 0.86, *p* = 0.001) and advantaged profiles (*OR* = 0.82, *p* < 0.001). Left‐behind status significantly increased the odds of belonging to the advantaged profile (OR = 1.90, *p* < 0.001) but did not predict membership in the maladaptive profile.

**TABLE 6:** Multinomial logistic regression predicting profile membership (Reference: Adaptive Profile).

Predictor	Maladaptive vs. Adaptive		Advantaged vs. Adaptive	
	OR [95% CI]	*p*	OR [95% CI]	*P*
Gender (ref: male)	1.007 [0.761, 1.334]	0.959	**0.754 [0.603, 0.942]**	**0.013**
Grade	**0.862 [0.790, 0.940]**	**0.001**	**0.815 [0.760, 0.874]**	**< 0.001**
Left‐behind (ref: no)	1.236 [0.849, 1.799]	0.274	**1.898 [1.374, 2.622]**	**< 0.001**

*Note*: Bold indicates p < 0.05. Gender and left‐behind coding follow the original questionnaire categories.

### 3.6 Interactive Effect of Psychological Capital and Academic Burnout on Short‐Video Addiction

To test whether psychological capital moderates the relationship between academic burnout and short‐video addiction, a multiple linear regression was performed with short‐video addiction as the outcome variable and psychological capital, academic burnout, and their interaction as predictors (see Table 7). Results showed a significant main effect of academic burnout (*B* = 0.225, *p* < 0.001) and a significant interaction effect (*B* = 0.035, *p* = 0.005), while the main effect of psychological capital was not significant (*p* = 0.709). The positive interaction indicates that the positive association between academic burnout and short‐video addiction strengthens as psychological capital increases.

**TABLE 7:** Interactive effect of psychological capital and academic burnout on Short‐Video Addiction.

Predictor	B	SE	*t*	*p*
Intercept	2.637	0.018	148.09	< 0.001
z_PC	0.008	0.02	0.37	0.709
z_AB	0.225	0.02	11.13	< 0.001
**z_PC × z_AB**	**0.035**	**0.012**	**2.81**	**0.005**

Abbreviations:; AB = Academic Burnout, PC = Psychological Capital, SVA = Short‐Video Addiction. R^2^ = 0.096, Adjusted R^2^ = 0.094, F (3, 1715) = 60.66, p < 0.001. ΔR^2^ for the interaction term = 0.004.

**FIGURE 2:**
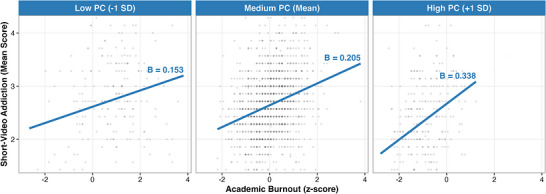
The moderating role of psychological capital in the relationship between academic burnout and short‐video addiction. Simple slopes are plotted at low (−1 SD), mean (0), and high (+1 SD) levels of psychological capital.

The interaction pattern is visualized in Figure 2. Simple slopes analysis revealed that the association between academic burnout and short‐video addiction was significant across all levels of psychological capital: at low (−1 SD), mean (0), and high (+1 SD) levels, the slopes were 0.19, 0.22, and 0.26, respectively (all *p* < 0.05). The Johnson−Neyman procedure indicated that the slope of academic burnout was significant for all observed values of psychological capital (range: −3.73 to 2.75). This indicates that while academic burnout consistently predicts addiction, the predictive strength modestly increases with higher psychological capital.

**FIGURE 3:**
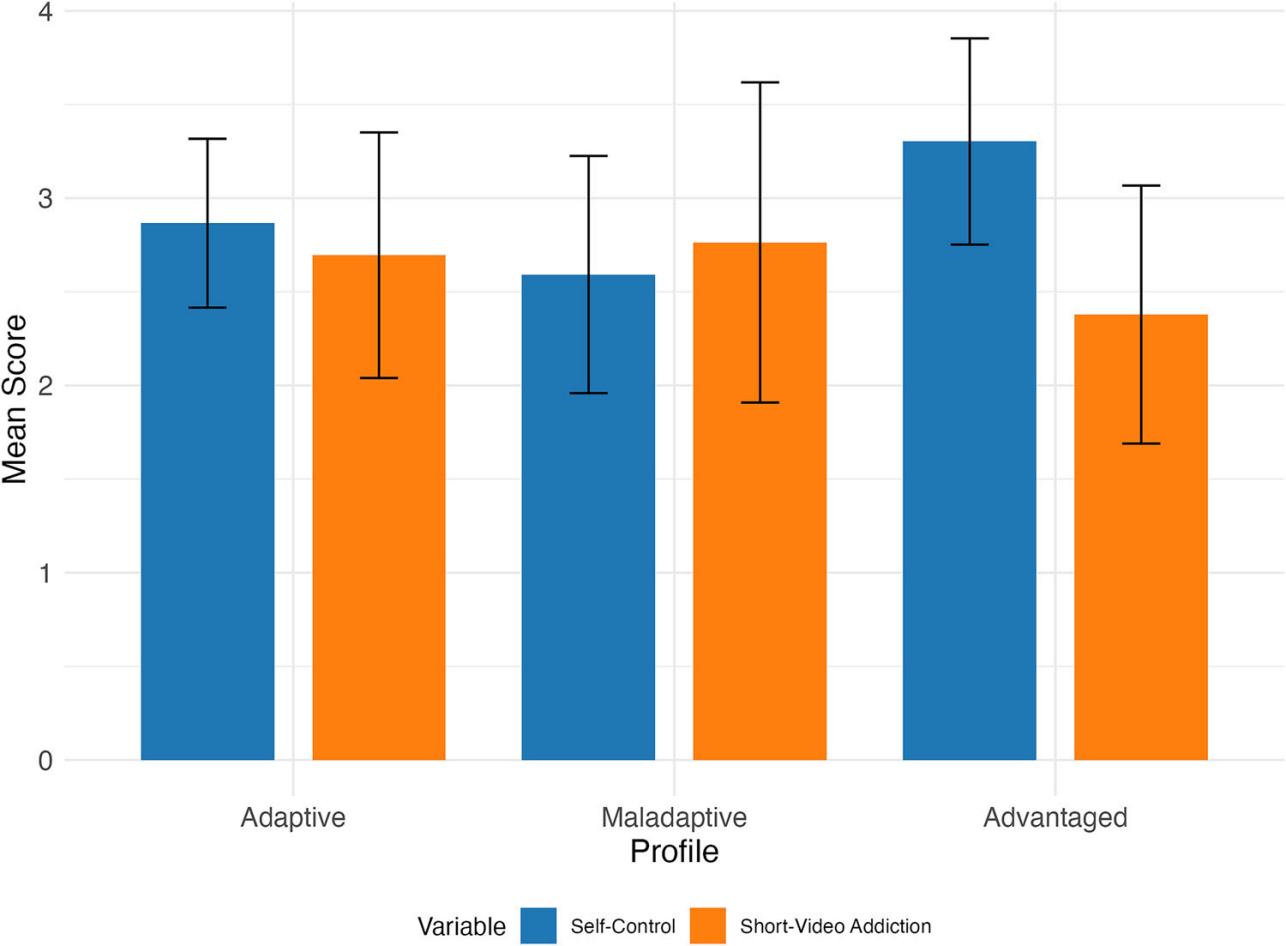
Mean scores of self‐control and short‐video addiction across the three latent profiles. Error bars indicate ±1 SD. ***p < 0.001.

## 4 Discussion

The present study employed a person‐centered approach to examine the heterogeneity of adolescents’ stress–resource profiles, operationalized by psychological capital and academic burnout, and further explored differences in self‐control and short‐video addiction across these profiles, along with predictors of profile membership and the interactive effect of psychological capital and academic burnout on addiction. The findings extend previous variable‐centered research by revealing three distinct profiles (Adaptive, 60.7%; Maladaptive, 12.9%; Advantaged, 26.4%), each characterized by unique combinations of psychological capital and academic burnout. These profiles showed significant differences in self‐control and short‐video addiction, and further analyses revealed that gender, grade, and left‐behind status predicted profile membership and that psychological capital moderated the relationship between academic burnout and short‐video addiction.

### 4.1 Heterogeneity in Stress–Resource Profiles

Consistent with Hypothesis 1, LPA identified three distinct subgroups among adolescents based on psychological capital and academic burnout. The Adaptive profile (57.6%) represented the majority, with both indicators near the average level. The Maladaptive profile (14.5%) was characterized by very low psychological capital and very high academic burnout, reflecting a severe resource deficit under high stress. The Advantaged profile (27.9%) showed the opposite pattern: high psychological capital and low academic burnout, indicating a resource‐rich, low‐stress state.

These findings align with the Conservation of Resources (COR) theory (Hobfoll 1989), which posits that individuals with resource deficits are more vulnerable to stress and maladaptive outcomes (a resource loss spiral), while those with abundant resources are better equipped to cope (a resource gain spiral). The identification of a sizable maladaptive subgroup highlights the importance of early detection and intervention for adolescents who lack psychological resources while experiencing high academic pressure. Moreover, the existence of an advantaged subgroup suggests that some adolescents successfully maintain high psychological capital despite low burnout, possibly due to effective coping strategies or supportive environments (Wang et al. 2025).

Compared to previous person‐centered studies among teachers (Zeng et al. 2021), which identified three profiles of work–family balance (contradictory, balanced, and active), the present study extends this typology to the adolescent context. While the adaptive and maladaptive profiles parallel the “balanced” and “contradictory” types, the advantaged profile is particularly notable as it captures adolescents who thrive academically and psychologically—a group that has been understudied in addiction research.

### 4.2 Self‐Control and Short‐Video Addiction Across Profiles

In support of Hypothesis 2, significant differences in self‐control and short‐video addiction were found across the three profiles. The Advantaged profile exhibited the highest self‐control and the lowest addiction levels, whereas the Maladaptive profile showed the lowest self‐control. However, post‐hoc comparisons indicated that the Adaptive and Maladaptive profiles did not differ significantly in short‐video addiction (p = 0.351); both groups showed significantly higher addiction than the Advantaged profile. This pattern suggests that self‐control serves as a key protective factor against addiction, but its protective effects may be most pronounced among those with favorable stress–resource conditions.

Interestingly, the Maladaptive and Adaptive profiles did not differ in short‐video addiction severity, despite the Maladaptive profile having much lower psychological capital and higher burnout. This indicates that high stress and low resources do not inevitably lead to addiction if self‐control remains at a moderate level. Conversely, the Advantaged profile's superior self‐control appears to effectively buffer against addiction. These findings refine our understanding of the interplay between stress, resources, and self‐regulation: self‐control may act as a “gatekeeper” that determines whether resource deficits translate into addictive behaviors. This pattern aligns with findings that self‐control directly predicts addiction severity (He et al. 2024; Miedzobrodzka et al. 2024), but further reveals that its protective effects vary across stress–resource profiles.

### 4.3 Predictors of Profile Membership

Consistent with Hypothesis 3, demographic factors significantly predicted profile membership. Gender: Female adolescents were less likely than males to belong to the Advantaged profile, suggesting that male adolescents may be more likely to report high psychological capital and low burnout. This aligns with previous research indicating gender differences in coping styles and resource utilization (Zhao et al. 2019). Grade: Higher grade levels were associated with lower odds of being in both Maladaptive and Advantaged profiles, indicating that adolescents become more likely to belong to the Adaptive profile as they advance in school. This may reflect a developmental trend toward greater stability in stress–resource balance or increased school‐related pressures that homogenize experiences. Left‐behind status: Notably, left‐behind adolescents were more likely to belong to the Advantaged profile. This counterintuitive finding warrants careful interpretation. One plausible explanation is that left‐behind children often develop greater independence, resilience, and reliance on social support networks outside the family (Wang et al. 2025; Yang et al. 2023), which may enhance their psychological capital and reduce academic burnout. Alternatively, this finding may reflect selection bias or regional characteristics of the sample (e.g., the presence of supportive extended family or community resources). Given that the majority of left‐behind adolescents in this study were from rural areas with strong kinship networks, their experiences may differ from those in urban or more isolated contexts. Future research should replicate this finding with diverse samples and include more nuanced measures of left‐behind experiences (e.g., duration of separation and caregiver quality).

### 4.4 Interactive Effect of Psychological Capital and Academic Burnout on Short‐Video Addiction

Hypothesis 4 was supported: psychological capital significantly moderated the relationship between academic burnout and short‐video addiction. The positive interaction term indicates that the association between burnout and addiction becomes stronger as psychological capital increases. This finding is particularly intriguing and adds nuance to the prevailing view of psychological capital as a purely protective factor.

One plausible explanation, grounded in COR theory, is that adolescents with high psychological capital possess greater cognitive and emotional resources, which may paradoxically lead them to engage in strategic escapism—using short videos as a deliberate, controlled coping mechanism when faced with burnout (Hobfoll 1989). When such individuals experience high burnout, they may actively seek out short videos to restore positive affect or regain a sense of control. However, given the highly engaging, algorithm‐driven nature of short‐video platforms (Huang et al. 2023), this intentional use can escalate into habitual overuse, thereby strengthening the burnout–addiction link. In contrast, adolescents with low psychological capital may lack the agency or motivation to use short videos strategically, resulting in a weaker burnout–addiction association. This “double‐edged sword” interpretation echoes recent findings that certain psychological strengths can amplify risk under adverse conditions (Luthans et al. 2007).

Alternatively, the interaction may reflect a reverse causality possibility: adolescents with high psychological capital may use short videos as a tool for social connection or achievement, which, under conditions of burnout, becomes maladaptive. Future longitudinal research is needed to disentangle these mechanisms.

It is also worth noting that the effect size of this interaction was modest (*R* ^2^ = 0.096), suggesting that while statistically significant, the interactive effect explains a relatively small portion of variance in addiction. This highlights the need for future research to incorporate additional variables (e.g., family environment, peer influence, and school climate) to more fully explain the complex pathways to short‐video addiction.

Simple slopes analysis further showed that the association between burnout and addiction remained significant across all levels of psychological capital (slopes: 0.19, 0.22, and 0.26 at low, mean, and high levels of psychological capital, respectively; all *p* < 0.05). The Johnson–Neyman procedure confirmed that the slope was significant for all observed values of psychological capital (range: −3.73 to 2.75). Thus, while the interaction is statistically detectable, its practical significance is limited, and the results should be interpreted with caution.

### 4.5 Theoretical Contributions and Practical Implications

This study makes several theoretical contributions. First, it is among the first to apply person‐centered LPA to adolescent short‐video addiction, revealing three distinct stress and resource profiles (Adaptive, Maladaptive, and Advantaged) that challenge the one‐size‐fits‐all approach of previous variable‐centered research. Second, it clarifies the role of self‐control: rather than functioning as a universal mediator, self‐control levels vary meaningfully across profiles, suggesting that interventions should be tailored accordingly. Third, the positive interaction between psychological capital and academic burnout on addiction challenges the assumption that psychological capital is universally protective, underscoring the need for more complex models of resource–stress interactions.

Tentatively, for maladaptive adolescents, interventions should prioritize building psychological capital (e.g., resilience training) and reducing academic burnout (e.g., stress management). School‐based programs like the HERO intervention (Heikkila et al. 2024) show promise. For advantaged adolescents, preventive programs should focus on maintaining healthy short‐video use habits. At the school level, fostering positive climates and providing mental health resources could help shift adolescents toward adaptive or advantaged profiles.

### 4.6 Limitations and Future Directions

Several limitations should be acknowledged. First, the cross‐sectional design precludes causal inferences; future longitudinal studies are needed to examine how profiles evolve over time. Second, data were collected from middle school students in five Chinese provinces, limiting generalizability to other cultural contexts or age groups. Third, self‐report measures may be subject to social desirability bias; future research could incorporate peer or teacher reports. Fourth, although the interaction effect was significant, its modest effect size suggests that other important variables (e.g., family environment and peer influence) should be included in future models. Fifth, the Entropy value (0.368) for the 3‐profile LPA solution was modest, indicating some overlap between profiles. This is likely due to the high correlation between psychological capital and academic burnout (*r* = −0.566). Future research should consider using additional indicators or subdimensions to improve classification accuracy. Sixth, due to the anonymous nature of data collection, school‐ or province‐level identifiers were not available; thus, clustering effects could not be statistically adjusted. Finally, the counterintuitive finding that left‐behind adolescents were more likely to be in the Advantaged profile warrants further investigation with more nuanced measures of left‐behind experiences (e.g., duration and caregiver quality).

## 5 Conclusion

This study employed a person‐centered approach to examine the heterogeneity of adolescents’ stress–resource profiles, as defined by psychological capital and academic burnout, and their associations with self‐control and short‐video addiction. Three distinct profiles were identified: Adaptive (57.6%, near‐average levels of both indicators), Maladaptive (14.5%, very low psychological capital and very high academic burnout), and Advantaged (27.9%, very high psychological capital and very low academic burnout). The Advantaged profile exhibited the highest self‐control and the lowest short‐video addiction, whereas the Maladaptive profile showed the lowest self‐control. Post‐hoc comparisons indicated that the Adaptive and Maladaptive profiles did not differ significantly in short‐video addiction, but both showed higher addiction than the Advantaged profile. Female adolescents were less likely than males to belong to the Advantaged profile, and higher grade levels reduced the likelihood of belonging to both Maladaptive and Advantaged profiles. Notably, left‐behind adolescents were more likely to be in the Advantaged profile, suggesting potential resilience mechanisms that warrant further investigation. Furthermore, psychological capital positively moderated the relationship between academic burnout and short‐video addiction, indicating that the association between burnout and addiction becomes stronger as psychological capital increases—a finding that challenges the view of psychological capital as universally protective.

The findings underscore the theoretical value of person‐centered methods in uncovering hidden heterogeneity and have practical implications for targeted prevention and intervention programs. Future longitudinal and cross‐cultural studies are needed to validate these profiles and elucidate the dynamic interplay of stress, resources, and self‐control in adolescent addictive behaviors.

## Author Contributions

All authors have made significant contributions to the work of the article. Lin and Lan are mainly responsible for the conceptualization and methodology. Lan is in charge of project management (ORCID: 0000‐0002‐9982‐6288). Liu H.Q. and Cui provided the funds. Lin, Lan, Xiong, Liu S.Y., Liu H., Cui, Guo and Wang were responsible for data investigation and analysis, as well as original manuscript writing and revision.

## Funding

This work was supported by the “Sailing” Plan Project of High‐level Talents in Yibin University (No. 2023QH29), the Research on the Localization Development Model of Township Social Work Service Stations in Southwest Ethnic Areas (No. 22BSH126), and the Outstanding Doctoral Talent Support Program of Guangxi Medical University.

## Ethics Statement

This study was approved by the Human Research Ethics Committee of Yibin University, China (2023101001E). All methods were carried out in accordance with the Declaration of Helsinki and approved by the aforementioned ethics committee. Before conducting the investigation, the researchers had explained the purpose and procedures to the participants. The participants and their guardians were informed and agreed to participate in this study.

## Consent

All authors have read and approved the final manuscript and consent to its publication.

## Conflicts of Interest

The authors declare no conflicts of interest.

## Data Availability

All data generated or analyzed during this study are included in this published article.
